# Impact of an Algorithm to Triage Patients Discharged From the Emergency Department With Blood Cultures Positive for *Staphylococcus aureus* or Coagulase-Negative *Staphylococcus*

**DOI:** 10.1016/j.acepjo.2024.100010

**Published:** 2025-01-10

**Authors:** Amy Mackowiak, Ethan Brenneman, Thomas Holland, Hui-Jie Lee, Justin Jones, Elizabeth Keil, Jennifer Mando, Rebecca Theophanous, Rachel Toler, Rebekah Moehring, Rebekah Wrenn

**Affiliations:** 1 Department of Pharmacy, Duke University Hospital, Durham, North Carolina, USA; 2 Division of Infectious Diseases, Duke University School of Medicine, Durham, North Carolina, USA; 3 Biostatistics & Bioinformatics, Duke University School of Medicine, Durham, North Carolina, USA; 4 Department of Pharmacy, Duke Raleigh Hospital, Raleigh, North Carolina, USA; 5 Emergency Medicine, Duke University School of Medicine, Durham, North Carolina, USA; 6 Department of Pharmacy, Duke Regional Hospital, Durham, North Carolina, USA

**Keywords:** emergency service, hospital, blood culture, Staphylococcus aureus, Staphylococcus, algorithm, patient discharge

## Abstract

**Objectives:**

Blood cultures obtained in the emergency department (ED) may become positive after discharge. Healthcare professionals must determine if these results represent true infection or a likely contaminant. An institutional algorithm was developed to assist with healthcare professional response to positive blood cultures for *S. aureus* and coagulase-negative staphylococci (CoNS) in these situations.

**Methods:**

We conducted a single system, multisite cohort study comparing before and after implementation of an ED decision-making algorithm from November 2022 to December 2023. Adults were included if they were discharged from the ED before blood cultures became positive for *Staphylococcus* species. The primary outcome was the difference in rates of patients called back to the ED pre- and postalgorithm implementation. Secondary endpoints evaluated algorithm adherence and safety.

**Results:**

A total of 253 patients, 188 pre- and 65 postimplementation, were enrolled. There was a 7.3% reduction in patients called back to the ED after algorithm implementation (95% CI [−21.1 to 6.3], *P* = .3). Algorithm adherence after implementation was 84.6% with a difference in actual and algorithm-based callback rates of 4.6%. After algorithm implementation, no patients deemed to have a contaminant experienced an infectious-related safety event.

**Conclusions:**

This time-saving algorithm was well received by our ED professionals and served as a helpful tool in safely and effectively triaging patients who had positive blood cultures for *Staphylococcus* species after discharge to determine who should be called back for further evaluation. There was a nonstatistically significant but clinically meaningful reduction in callback rates. Postimplementation algorithm adherence was high, and the majority of callback decisions were appropriate.

**The Bottom Line**

Patients may be discharged from the emergency department (ED) prior to blood culture results. If these cultures become positive, medical professionals must determine who should be called back for further evaluation. We developed a time-saving algorithm that was well received by our ED professionals and served as a helpful tool in safely and effectively triaging patients who had positive blood cultures for *Staphylococcus* species after discharge.

## 1 Introduction

### 1.1 Background

Emergency department (ED) workflows often require early diagnosis and treatment with minimal patient information as part of a constantly evolving and rapid decision-making process. Thus, patients are sometimes discharged from the ED before the final blood culture results, which presents a clinical and logistical challenge. If blood cultures become positive after discharge, an ED professional must carefully evaluate these results to determine the next steps in caring for the patient. Patients brought back to the ED may receive intravenous antibiotic therapy, have repeat blood cultures drawn, and utilize significant ED resources. Up to 55% of positive cultures may be contaminants, and calling these patients back to the ED can cause undue burden to medical staff, resources, and the patient if no treatment or further evaluation is needed.^1^ Staphylococci are the most commonly identified pathogens in both hospital-acquired and community-onset bloodstream infections.^2^ *Staphylococcus aureus* isolated from the bloodstream should always be evaluated carefully as it is exceedingly uncommon to be deemed a contaminant.^3^ Coagulase-negative staphylococci (CoNS) in blood cultures, however, are often contaminants and may not require any treatment.^4^^,^^5^ True bacteremia caused by CoNS does warrant treatment and return to the ED.^6^

### 1.2 Importance

Holland et al^6^ sought to compare a standardized algorithm for the categorization and determination of treatment duration for staphylococcal bacteremia. Algorithm-based therapy reduced mean antibiotic duration compared with usual practice in patients who had simple or uncomplicated bacteremia, with the largest difference noted in patients who had CoNS. This study highlights that in patients with likely contamination with CoNS, no treatment was warranted, and that patients did not need to return to an ED. A decision-making algorithm to help healthcare professionals determine if staphylococci in a blood culture represents true infection or contamination could streamline ED workflow and minimize unnecessary returns to the ED while advising return for true infection.

### 1.3 Goals of This Investigation

There have been studies focused on the decision-making process for drawing blood cultures in the ED, but there is a lack of literature evaluating standardized ED-based algorithms for assessing patients who have been discharged home with pending blood cultures.^7^^,^^8^ We hypothesized that the implementation of a decision-making algorithm would reduce patient callbacks without increasing adverse events.

## 2 Methods

### 2.1 Study Design and Study Setting

We performed a pre/postintervention study to compare outcomes before and after the implementation of an ED patient callback decision-making algorithm in a single health care system. In phase I (prealgorithm implementation) from January 1, 2019 to November 2, 2022, we retrospectively applied the algorithm to determine baseline practice patterns. Then, the algorithm was implemented across our health care system from November 3, 2022 to December 1, 2022. During implementation, physicians, advanced practice professionals (APPs), and resource nurses were educated about the algorithm, and the use of the algorithm was integrated into existing workflows. Phase II (postalgorithm implementation) involved a retrospective review of algorithm utilization from December 2022 to midJuly 2023 and a prospective review from midJuly 2023 to December 2023. The prospective component also involved direct and real-time feedback by the implementation team to the treating physician or APP and resource nurses via Electronic Health Record (EHR) secure chat when deviations from the algorithm occurred. Additionally, follow-up education was repeated during the postalgorithm implementation period in June 2023. This study was approved by our hospital’s Institutional Review Board as exempt and reported according to the Strengthening the Reporting of Observational Studies in Epidemiology cohort study guidelines.

Our study setting consisted of 1 academic and 2 community hospital-affiliated EDs with an average combined volume of 185,000 ED visits per year. Our hospital microbiology laboratories utilize Biofire blood culture identification panels for rapid identification, and our health care system utilizes a blood culture collection algorithm to standardize decision-making for drawing blood cultures.^9^ This algorithm considers a patient’s immunocompromised status, clinical instability, and pretest probability of bacteremia. At our institution, resource nurses are alerted to positive blood cultures that result after discharge via EHR in-basket messages, which are then relayed to the staffing ED physician or APP, who make the decision whether or not the patient should be called back for further evaluation. This recommendation is communicated by telephone to the patient and documented in the patient chart. If the patient is unable to be reached after 3 attempts, a letter is sent to their address.

### 2.2 Selection of Participants

**Figure 1:**
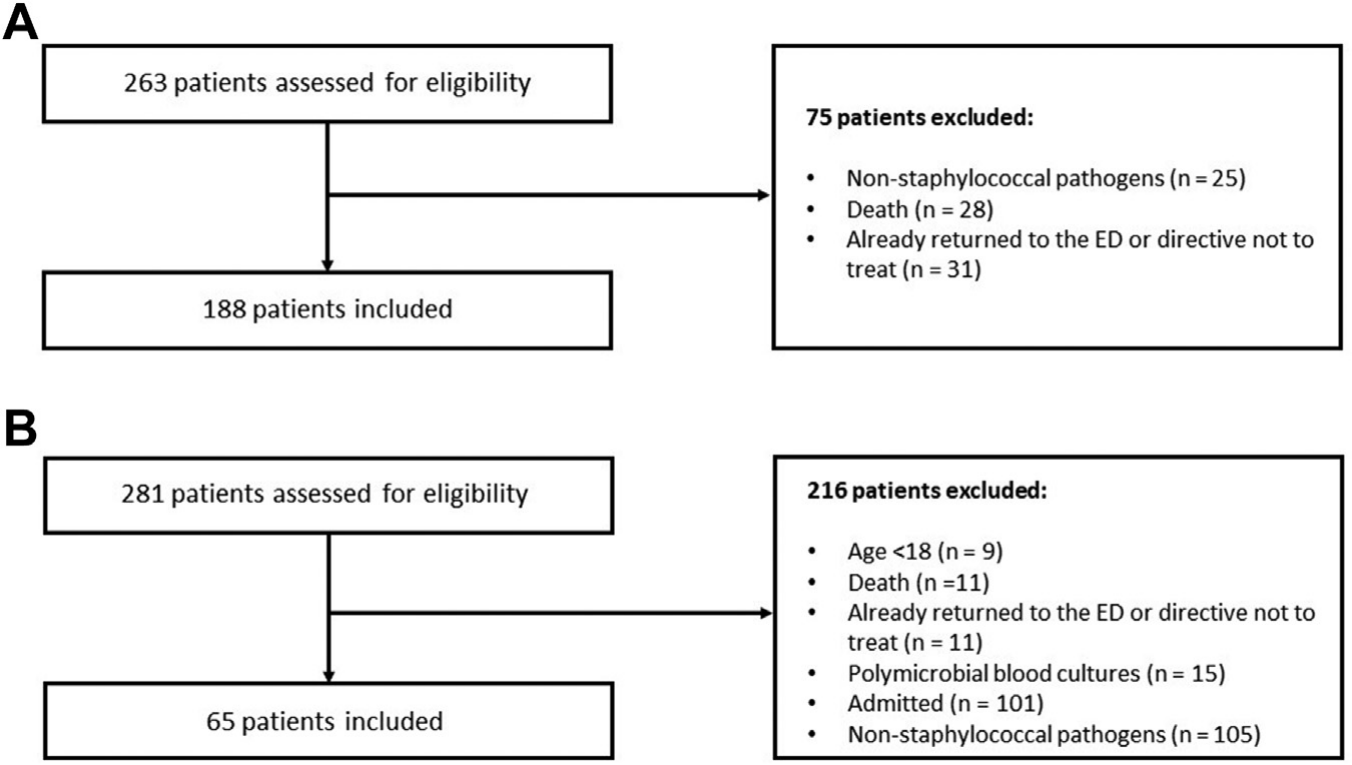
A, Methods flow diagram prealgorithm implementation (patients may have multiple exclusion reasons). B, Methods flow diagram postalgorithm implementation (patients may have multiple exclusion reasons).

We included patients who were aged ≥18 years, had visited one of our health care system’s affiliated EDs, and had blood cultures with growth for *S. aureus* or CoNS that resulted after discharge from the ED. Exclusion criteria were the following: growth of nonstaphylococcal pathogens in blood cultures, polymicrobial bloodstream infections (unless it was because of numerous CoNS species), mortality prior to blood culture growth, an advanced directive to not treat infections, or if patients had already returned to the ED or were admitted to the hospital when blood cultures resulted with growth **(**Fig 1).

### 2.3 Intervention

**Figure 2:**
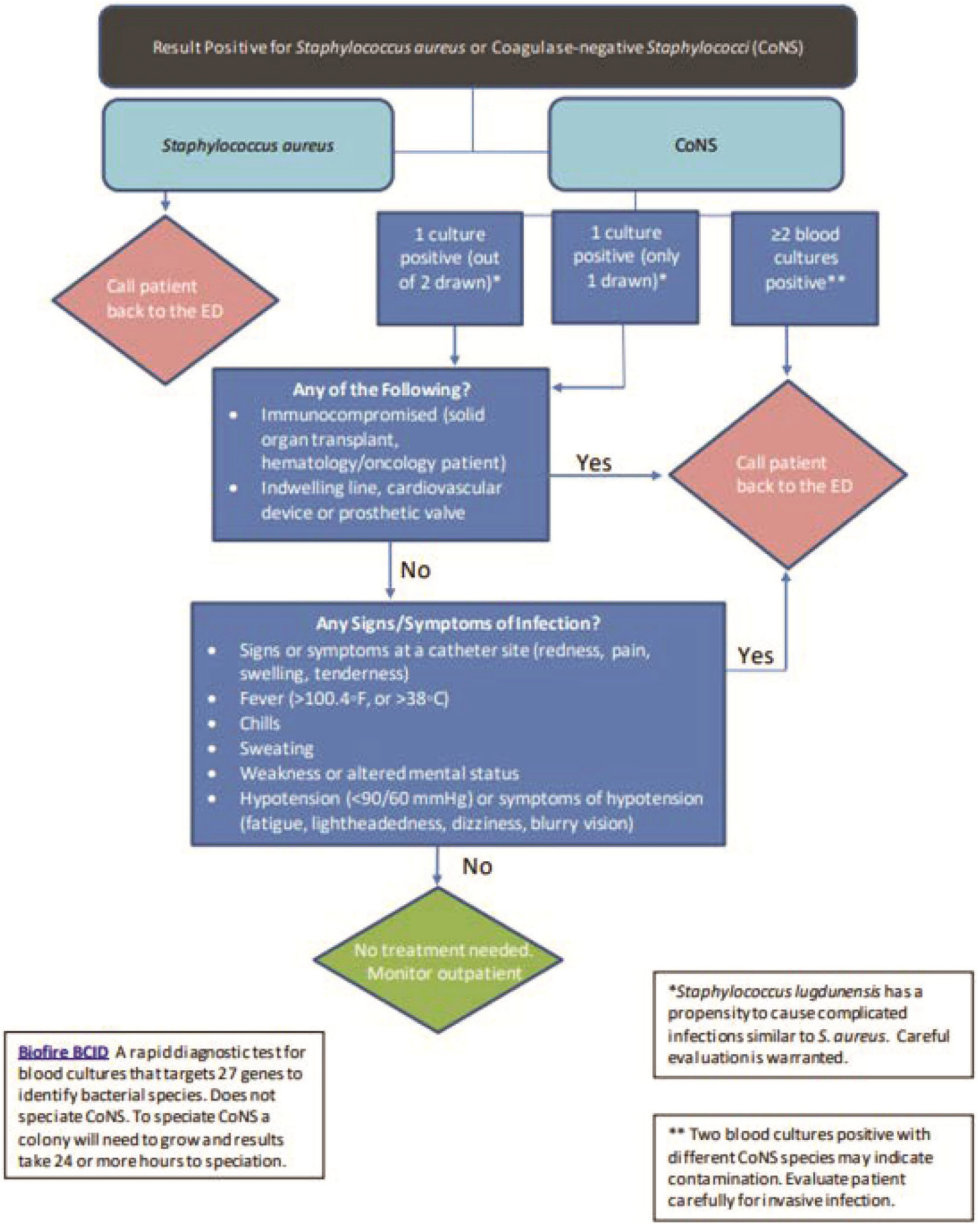
Callback algorithm for positive blood cultures with *Staphylococci.*

We developed an algorithm to assist physicians and APPs in responding to positive blood cultures for *S. aureus* and CoNS in patients who have been discharged from the ED (Fig 2). The algorithm identifies which patients need to return to the ED and are therefore called back by our ED staff for further evaluation and which patients do not need to return to the ED because of the likelihood for blood culture contamination with CoNS using criteria from the Holland et al^6^ study as well as other published algorithms.^10^

### 2.4 Data Collection and Measurements

Potential study subjects were identified using Epic Clarity and hospital EHR reports. Epic Clarity is a normalized relational database that is housed in Oracle and used for analytical reporting that stores EHR data on a daily basis. To capture patients for the prospective study review, an EHR patient list was built that included patients with positive blood cultures and recent discharge from the ED. For enrolled patients, data were gathered automatically via Clarity and manual EHR chart review. Study data were managed using a secure, web-based software platform (Research Electronic Data Capture or REDCap) as electronic data capture tools hosted at the main institution.^11^^,^^12^ For retrospective data collection, Clarity was used to collect microbiology culture results, baseline patient demographics, and location and timing of the index ED visit. Data on ED response to blood cultures, 30- and 60-day readmissions (to the ED or inpatient) mortality, and antibiotic regimens were collected manually. A manual EHR chart review by 1 primary reviewer (A.M.) was utilized for all components of prospective data collection. The callback algorithm was manually applied to each patient, and an “appropriate response” was determined using the algorithm’s flowchart by a member of the implementation team (A.M. and E.B.). For example, for a patient with *S. aureus* growing in the blood cultures, the “appropriate response” was to instruct the patient to return to the ED. As another example, a patient who had 1 of 2 CoNS growth, was without indwelling lines or immunosuppression, and was asymptomatic after discharge should not be instructed to return because this patient would be low risk and most likely a contaminant based on the algorithm and prior literature. Every patient whom the algorithm deemed to not need a callback and who had positive repeat blood cultures or a possible infection-related adverse outcome was reviewed by 4 ED or infectious diseases investigators (2 PharmDs [A.M., E.B., or R.W.] and 2 MDs [T.H. and R.M.]) to determine whether the patient met the criteria for infection-related adverse outcomes.

### 2.5 Outcomes

Our primary outcome was the rate of patients called back to the ED pre- versus postimplementation of the ED callback algorithm. Secondary outcomes included ED physician and APP algorithm adherence rate pre- and postimplementation, 30- and 60-day infection-related readmission, ED visits and mortality, and the rates of positive follow-up blood cultures which served as another surrogate for true infection. Infection-related ED visits, admissions, and mortality were defined as a visit/encounter deemed to be related to the index blood cultures, such as a positive culture for the same species of CoNS requiring treatment or a visit related to potential sequelae from staphylococcal infection (ie, drainage from a wound site). The definition was designed to exclude ED visits and admissions for unrelated infections such as gram-negative urinary tract infections. ED visit and readmission outcomes excluded the initial return visit for patients called back to the ED.

### 2.6 Data Analyses

We described patient and encounter characteristics using summary statistics. The rates of callback to the ED for both the pre- and postimplementation period were defined as the number of patients told to return to the ED (callback) divided by the total number of patients with positive blood cultures and 95% Wilson score binomial confidence intervals were estimated. The difference in rates between the 2 periods was estimated with 95% CIs using Newcombe’s method. A 2-sample *z*-test for binomial proportions with unpooled variance was used to compare the 2 rates.

The rate of callback to ED based on the algorithm was calculated similarly. The difference in callback rate and the per-algorithm callback rate for each period was estimated with 95% CI using the Newcombe square-and-add approach and compared using an Asymptotic McNemar’s test for paired binomial proportions because each encounter was evaluated twice for actual and per-algorithm callback.^13^ All statistical analyses were conducted in R 4.2.2 (R Core Team). The statistical test was 2-sided with a 0.05 level of significance.

## 3 Results

### 3.1 Characteristics of Study Subjects

A total of 253 patients were included in the study, 188 preimplementation and 65 postimplementation. The patient population was reflective of the overall academic/community health system population and baseline characteristics were overall well-balanced between the cohorts. There was a slightly higher proportion of patients who had immunocompromising conditions in the prealgorithm group and more patients who had prosthetic material in the postalgorithm group. Although only a small portion of patients included in the study had significant immunosuppression, this was likely because those patients were more frequently admitted when initially seen in the ED (Table 1).

**Table 1:** Patient characteristics from index ED encounter.

Characteristic	Preimplementation (N = 188)	Postimplementation (N = 65)	Total (N = 253)
Age (y), median (Q1 and Q3)	59.3 (45.0, 72.1)	57.9 (37.8, 72.8)	58.9 (43.4, 72.6)
Sex – female, n (%)	97 (51.6)	35 (53.8)	132 (52.2)
Weight (kg), median (Q1 and Q3)	85.6 (70.3, 104.3)	77.7 (63.5, 100.5)	83.5 (68.7, 103.6)
Calculated BMI (kg/m^2^), median (Q1 and Q3)	30.2 (25.9, 37.5)	28.4 (22.2, 37.2)	29.5 (24.8, 37.4)
Race, n (%)			
Black or African American	90 (47.9)	31 (47.7)	121 (47.8)
Caucasian/White	90 (47.9)	30 (46.2)	120 (47.4)
Asian	3 (1.6)	0 (0.0)	3 (1.2)
Other	4 (2.1)	1 (1.5)	5 (2.0)
Not Reported/decline	1 (0.5)	3 (4.6)	4 (1.6)
Comorbidities, n (%)			
Diabetes	25 (13.3)	16 (24.6)	41 (16.2)
Chronic kidney disease	38 (20.2)	13 (20.0)	51 (20.2)
Chronic liver disease	9 (4.8)	3 (4.6)	12 (4.7)
Surgery in last 30 days	12 (6.4)	3 (4.6)	15 (5.9)
Trauma in last 30 days	10 (5.3)	0 (0.0)	10 (4.0)
Injection drug use	8 (4.3)	4 (6.2)	12 (4.7)
Preexisting valvular heart disease	3 (1.6)	0 (0.0)	3 (1.2)
Prior *Staphylococcal aureus* infection within past year (any infection)	17 (9.0)	3 (4.6)	20 (7.9)
Prior *Staphylococcal aureus* bloodstream infection within past year	9 (4.8)	1 (1.5)	10 (4.0)
Prosthetic material present, n (%)			
Indwelling line	20 (10.6)	12 (18.5)	32 (12.6)
Cardiovascular valve	7 (3.7)	1 (1.5)	8 (3.2)
Prosthetic valve	3 (1.6)	0 (0.0)	3 (1.2)
Other	4 (2.1)	1 (1.5)	5 (2.0)
None	157 (83.5)	51 (78.5)	208 (82.2)
Immunocompromising condition, n (%)			
Chronic steroids	2 (1.1)	0 (0.0)	2 (0.8)
Solid organ transplant	6 (3.2)	4 (6.2)	10 (4.0)
Hematology/oncology condition	4 (2.1)	7 (10.8)	11 (4.3)
Other immunomodulatory agent	12 (6.4)	0 (0.0)	12 (4.7)
None	166 (88.3)	54 (83.1)	220 (87.0)
No. of index cultures drawn, median (Q1 and Q3)	2 (2, 2)	2 (2, 2)	2 (2, 2)
No. of index cultures with growth, n (%)			
1	154 (81.9)	54 (83.1)	208 (82.2)
2	34 (18.1)	11 (16.9)	45 (17.8)
Species identified			
CoNS (Not *S. lugdunensis*)	154 (81.9)	58 (89.2)	212 (83.8)
*S. lugdunensis*	0 (0.0)	1 (1.5)	1 (0.4)
*S. aureus*	36 (19.1)	6 (9.2)	42 (16.6)
Methicillin-resistant *S. aureus*	14 (7.4)	3 (4.6)	17 (6.7)
Methicillin-susceptible *S. aureus*	23 (12.2)	3 (4.6)	26 (10.3)
Time to identification: CoNS (h), median (Q1 and Q3)	30.8 (25.8, 40.8)	31.0 (25.1, 39.6)	30.9 (25.7, 40.4)
Time to identification: *S. aureus* (h), median (Q1 and Q3)	23.7 (19.6, 34.9)	17.9 (14.2, 36.9)	23.6 (18.9, 37.5)
Time from gram stain to species identification (h), median (Q1 and Q3)	1.6 (1.0, 2.4)	1.5 (0.0, 2.3)	1.6 (0.7, 2.4)
Time from gram stain results to first call (h), median (Q1 and Q3)	2.3 (0.7, 9.6)	6.0 (2.2, 20.0)	2.8 (0.9, 10.1)
Index symptoms, n (%)			
Fever	66 (35.1)	20 (30.8)	86 (34.0)
Chills	28 (14.9)	11 (16.9)	39 (15.4)
Sweating	6 (3.2)	2 (3.1)	8 (3.2)
Weakness	15 (8.0)	13 (20.0)	28 (11.1)
AMS	13 (6.9)	10 (15.4)	23 (9.1)
Hypotension	10 (5.3)	3 (4.6)	13 (5.1)
Symptoms at catheter site	3 (1.6)	4 (6.2)	7 (2.8)
Other	140 (74.5)	14 (21.5)	154 (60.9)
None	9 (4.8)	11 (16.9)	20 (7.9)
Patient Called			
Yes	165 (87.8%)	47 (72.3%)	212 (83.8%)
No	23 (12.2%)	18 (27.7%)	41 (16.2%)
Patient successfully contacted on first call			
Yes	131 (69.7%)	35 (53.8%)	166 (65.6%)
No	34 (18.1%)	12 (18.5%)	46 (18.2%)
Not called	23 (12.2%)	18 (27.7%)	41 (16.2%)
Patient successfully contacted (first or repeated calls)			
Yes	153 (81.4%)	37 (56.9%)	190 (75.1%)
No	12 (6.4%)	10 (15.4%)	22 (8.7%)
Not called	23 (12.2%)	18 (27.7%)	41 (16.2%)

### 3.2 Main Results

Overall, 118 (46.6%) patients, 86 (45.7%) preimplementation and 32 (49.2%) postimplementation, were determined to have met peralgorithm requirements for a callback to the ED based on patient characteristics or infectious symptoms during a wellness check. In actual practice, 115 (61.2%) preimplementation and 35 (53.9%) patients postimplementation were told to return to the ED. The rate of patients told to return to the ED was decreased by 7.3% from pre- to postalgorithm implementation (95% CI −21.1% to 6.3%; *P* = .3) (Table 2). “Adherence” to the algorithm before implementation was 68.6%, with a difference between the actual and the algorithm-based callback rates of 15.4% (95% CI 9.8% to 20.9%, *P* < .001). Conversely, the algorithm adherence postimplementation was 84.6%, with a difference in actual and algorithm-based callback rates of 4.6% (95% CI −5.6% to 14.6%, *P* = .55) (Table 3). The breakdown of positive cultures and callbacks by site can be seen in the Supplementary Appendix 1.

**Table 2:** Difference in the rate of patient callbacks and algorithm adherence pre- and postimplementation.

Outcome	Preimplementation	Postimplementation	Difference (Post–Pre)
Rate of patients told to return to ED, n/n(%); 95% CI	115/188 (61.2) 9 (54.1, 67.9)	35/65 (53.9) 9 (41.9, 65.4)	−7.3 9 (−21.1 to 6.3); *P* = .3^a^
Rate of adherence to the algorithm for all patients, n/n(%); 95% CI	129/188 (68.6) 9 (61.7, 74.8)	55/65 (84.6) 95% CI: (73.9, 91.4)	16% 9 (−3.7 to 25.7); *P* = .01^a^

a P-values were from 2-sample *z*-tests for binomial proportions with unpooled variance.

**Table 3:** Rates of algorithm adherence by organism grown in index blood culture pre- and post-implementation.

Outcomes, n/n (%)	Actual	Per Algorithm	Difference in rates (actual – per algorithm)	Newcombe 95% CI	*P*-value
Preimplementation period					
Rate of patients told to return to ED	115/188 (61.2)	86/188 (45.7)	29/188 (15.4)	(7.7, 22.8)	<.001^a^
Rate of patients with *S. aureus* told to return to ED	32/36 (88.9)	36/36 (100)	−4/36 (−11.1)		
Rate of patients with CoNS told to return to ED	85/154 (55.2)	52/154 (33.8)	33/154 (21.4)		
Postimplementation period					
Rate of patients told to return to ED	35/65 (53.85)	32/65 (49.23)	3/65 (4.62)	(−5.59 to 14.6)	.55^a^
Rate of patients with *S. aureus* told to return to ED	6/6 (100)	6/6 (100)	0/6 (0)		
Rate of patients with CoNS told to return to ED	29/58 (50)	25/58 (43.1)	4/58 (6.9)		
Rate of patients with *S. lugdunensis* told to return to ED	0/1 (0)	1/1 (100)	−1/1 (−100)		

ED, emergency department.

a *P*-values were from asymptotic McNemar’s tests for paired binomial proportions.

In the preimplementation period, 57 (30%) patients had discordant results between the actual callback decision and the algorithm decision compared with 11 (16.9%) patients in the postimplementation period. In the prospective portion of the postimplementation period, there was only 1 discrepancy between actual and per-algorithm care, where feedback had to be provided to the ED team. The largest group of patients with algorithm discrepancy were those with 1 of 2 CoNS growth on index blood cultures that were inappropriately called back (Table 3).

Of the 102 patients in the preimplementation period that the algorithm identified retrospectively as having a contaminant and not needing to return to the ED, there was only 1 (1.7%) patient with a staphylococcal infection-related ED visit or readmission within 60 days, which also resulted in an infection-related 60-day mortality event (Table 4). In the postimplementation period, 33 patients were identified as having a contaminant, none of which experienced an infectious-related safety event (Table 5). Of the 6 total patients with growth on repeat blood cultures, none were deemed to be true infections related to the index blood cultures after investigator review.

**Table 4:** Safety-related outcomes of the preimplementation period based on the ED response.

Outcome	Patients appropriately called back (N = 72)	Patients called back but should not have been (N = 43)	Patients not called back who should have been (N = 14)	Patients appropriately not called back (N = 59)	Total Patients (N = 188)
Callback visit resulting in admission	49 (68.1)	11 (25.6)	4 (28.6)	3 (5.1)	67 (35.6)
60-day infection related ED visit	2 (2.8)	1 (2.3)	1 (7.1)	1 (1.7)	5 (2.7)
60-day infection related admission	5 (6.9)	1 (2.3)	2 (14.3)	1 (1.7)	9 (4.8)
60-day mortality	5 (6.9)	2 (4.7)	0 (0.0)	3 (5.1)	10 (5.3)
60-day infection related mortality?	4 (5.6)	0 (0.0)	0 (0.0)	1 (1.7)	5 (2.7)

ED, emergency department.

**Table 5:** Safety-related outcomes of the postimplementation period based on the ED response.

Outcome	Patients appropriately called back N = 28	Patients called back but should not have been N = 7	Patients not called back who should have been N = 4	Patients appropriately not called back N = 26	Total N = 65
Callback visit resulting in admission, n (%)	12 (42.9)	0 (0.0)	0 (0.0)	0 (0.0)	12 (18.5)
60-d infection related ED visit, n (%)	0 (0.0)	0 (0.0)	0 (0.0)	0 (0.0)	0 (0.0)
60-d infection related admission, n (%)	1 (3.6)	0 (0.0)	0 (0.0)	0 (0.0)	1 (1.5)
60-d mortality	1 (3.6)	0 (0.0)	0 (0.0)	0 (0.0)	1 (1.5)
60-d infection related mortality? , n (%)	0 (0.0)	0 (0.0)	0 (0.0)	0 (0.0)	0 (0.0)

## 4 Limitations

This study had key limitations. Reviewers applied the algorithm retrospectively using only data from the EHR. There was potential for differences in the interpretation of algorithm implementation by reviewers and errors stemming from lack of documentation. To mitigate the risk of misinterpretation of infection-related adverse events for patients deemed to have a contaminant, cases were reviewed by a 4-member committee as previously described. Additionally, discharged patients who re-presented to an outside ED facility or hospital that did not share an EHR platform would have been missed. There is also the concern that the generalizability of our study may be limited by our patient cohort, as our academic center treats higher acuity and more complex patients than typical community hospitals or EDs. However, the inclusion of 3 separate hospital sites, including 2 community-affiliated hospitals, improves validity. Finally, the sample size in the postalgorithm group was small, less than half the size of our precohort, and we were unable to show that the reduced rates of ED callbacks were statistically significant. Nevertheless, we considered a difference of 7.3% as clinically meaningful, considering the ED resources saved.

## 5 Discussion

Between 1997 and 2016, ED visits have risen by more than 60% to around 146 million annually, and patients frequently have blood cultures drawn during those visits.^14^ It is critical to identify which discharged patients with blood cultures positive for CoNS can be deemed contaminants and do not warrant follow-up in order to reduce the number of total ED visits and the utilization of resources. Additionally, it is critical to identify and callback true infections for further evaluation. To our knowledge, this is the first study that has evaluated a standardized ED-based algorithm for assessing patients discharged home with pending blood cultures for *Staphylococcus* species.

During phase I of this study, implementation of the decision-making algorithm would have reduced the rate of discharged patients called back to the ED with staphylococci in blood cultures by 25% without leading to adverse safety outcomes. We observed a 7.3% (95% CI -21.1 to 6.3) reduction in patients called back to the ED after algorithm implementation combined across all sites. Although this number was not statistically significant, the reduction in patient callbacks is clinically significant for reducing ED volumes, expenditure of unnecessary resources, and improved patient-centered outcomes. Algorithm adherence was close to 85% in the postalgorithm period, and the majority of patients called back to the ED were appropriate. This increase in adherence from pre- to postalgorithm implementation was seen in each individual site as well, although there was an increase in callbacks among our 2 community-affiliated hospitals. This again highlights the importance of decision-making based on patient and infectious related characteristics to ensure patients who should be called back for further evaluation are told to do so while minimizing callbacks for likely contaminants to preserve ED resources. During the preimplementation phase, we learned that some callback decisions were made based on the gram stain instead of waiting for species identification. Both *S. aureus* and CoNS are indistinguishable on gram stains as “gram-positive cocci in clusters” yet appropriate clinical responses should be based on the pathogen and patient risks. Physicians, APPs, and nurses were educated on the importance of waiting for species identification. During postimplementation, there was increased compliance with waiting for species identification, which was reflected by an extension in the “time from gram stain to first call” by approximately 3 hours.

The algorithm focused only on staphylococci as they are common bacteria in blood cultures, and CoNS has clear population characteristics that identify patients with a high likelihood of representing a contaminant.^4^, ^5^, ^6^ Previous studies assessing CoNS as a contaminant mostly focused on hospitalized patients, whereas our study exclusively focused on EDs and outpatients.^6^^,^^10^ This may have been selected for patients most likely to have contaminants, as EDs have higher contamination rates than inpatient units, and patients were well enough to be discharged before cultures. In one study assessing a similar algorithm, patients without central lines and no SIRS criteria had a negative predictive value of 91.7% for infection.^10^ In comparison, only 1 patient in our population deemed to have a contaminant had an infection-related adverse event.

In line with the Holland et al^2^^,^^6^ trial, our study found that patients who had 1 out of 2 blood cultures for CoNS, did not have indwelling lines, significant immunosuppression, or symptoms of infection could be safely assumed to have blood culture contaminants and did not need to be treated with antibiotics. This study also highlights the importance of having all patients who have *S. aureus* in blood cultures seen by a healthcare professional as soon as possible. Patients with *S. aureus* were most likely to experience infection-related adverse outcomes and had the highest rates of positive repeat blood cultures. There were 4 patients with *S. aureus* in the preimplementation group who were not told to return, all of whom ended up being admitted to the hospital because of worsening infectious symptoms. The most common reason that these patients were not called back was their missing telephone and address information; therefore, resource nurses were unable to contact the patient with these results. All 6 patients with *S. aureus* on index blood cultures in the postalgorithm implementation group were appropriately called back to the ED.

In summary, the blood culture callback algorithm was simple and served as a quick decision-making tool for ED physicians, APPs, and nurses to triage patients with positive blood cultures for *S. aureus* or CoNS after ED discharge. There was a 7.3% callback reduction rate between pre- and postalgorithm implementation, with higher algorithm adherence in the postimplementation period (approximately 85%). No patients who experienced an infection-related safety event in the postalgorithm implementation period who were not called back for blood cultures were deemed a contaminant, suggesting that the blood culture algorithm reduced unnecessary ED callbacks without compromising patient safety.

## Author Contributions

AM, EB, EK, RW designed the study and supervised the study content and data collection. EB, JJ, JM, RT, and RW educated professionals on algorithm implementation. HL performed statistical analysis. AM, EB, TH, RM, and RW participated in blood culture and infection-related safety reviews. AM and EB drafted the manuscript. TH, HJ, JJ, EK, RT, JM, RM, and RW contributed to manuscript revisions and reviews.

## Funding and Support

By *JACEP* *Open* policy, all authors are required to disclose any and all commercial, financial, and other relationships in any way related to the subject of this article as per ICMJE conflict of interest guidelines (see www.icmje.org). The authors have stated that no such relationships exist.

## Conflict of Interest

All authors have affirmed they have no conflicts of interest to declare.

## References

[1] Schinkel M., Boerman A.W., Bennis F.C. (2022). Diagnostic stewardship for blood cultures in the emergency department: a multicenter validation and prospective evaluation of a machine learning prediction tool. EBioMedicine.

[2] Holland T.L., Arnold C., Fowler V.G. (2014). Clinical management of Staphylococcus aureus bacteremia: a review. JAMA.

[3] Mermel L.A., Allon M., Bouza E. (2009). Clinical practice guidelines for the diagnosis and management of intravascular catheter-related infection: 2009 update by the Infectious Diseases Society of America. Clin Infect Dis.

[4] Liu C., Bayer A., Cosgrove S.E., Infectious Diseases Society of America (2011). Clinical practice guidelines by the Infectious Diseases Society of America for the treatment of methicillin-resistant *Staphylococcus aureus* infections in adults and children. Clin Infect Dis.

[5] Weinstein M.P., Towns M.L., Quartey S.M. (1997). The clinical significance of positive blood cultures in the 1990s: a prospective comprehensive evaluation of the microbiology, epidemiology, and outcome of bacteremia and fungemia in adults. Clin Infect Dis.

[6] Holland T.L., Raad I., Boucher H.W. (2018). Effect of algorithm-based therapy vs usual care on clinical success and serious adverse events in patients with staphylococcal bacteremia: a randomized clinical trial. JAMA.

[7] Shapiro N.I., Wolfe R.E., Wright S.B., Moore R., Bates D.W. (2008). Who needs a blood culture? A prospectively derived and validated prediction rule. J Emerg Med.

[8] Theophanous R., Ramos J., Calland A.R. (2024). Blood culture algorithm implementation in emergency department patients as a diagnostic stewardship intervention. Am J Infect Control.

[9] The BioFire® FilmArray® Blood Culture Identification Panels. bioMérieux. Accessed May 2023. https://www.biofiredx.com/products/the-filmarray-panels/filmarraybcid/

[10] Elzi L., Babouee B., Vögeli N. (2012). How to discriminate contamination from bloodstream infection due to coagulase-negative staphylococci: a prospective study with 654 patients. Clin Microbiol Infect.

[11] Harris P.A., Taylor R., Thielke R., Payne J., Gonzalez N., Conde J.G. (2009). Research Electronic Data Capture (REDCap) --a metadata-driven methodology and workflow process for providing translational research informatics support. J Biomed Inform.

[12] Harris P.A., Taylor R., Minor B. (2019). The REDCap consortium: building an international community of software platform partners. J Biomed Inform.

[13] Fagerland M.W., Lydersen S., Laake P. (2014). Recommended tests and confidence intervals for paired binomial proportions. Stat Med.

[14] National hospital ambulatory medical care survey: 2016 emergency department summary tables. https://www.cdc.gov/nchs/data/nhamcs/web_tables/2022-nhamcs-ed-web-tables.pdf.

