# Supplementary material for: Impact of an Algorithm to Triage Patients Discharged From the Emergency Department With Blood Cultures Positive for Staphylococcus aureus or Coagulase-Negative Staphylococcus
Source: J Am Coll Emerg Physicians Open. 2025 Jan 10;6(1):100010. doi: 10.1016/j.acepjo.2024.100010 (PMC11852945; doi:10.1016/j.acepjo.2024.100010)
Supplement: Supplementary Appendix 1 [file mmc1.docx]

**Supplemental Material**

**Figure S1.**  Study Timeline


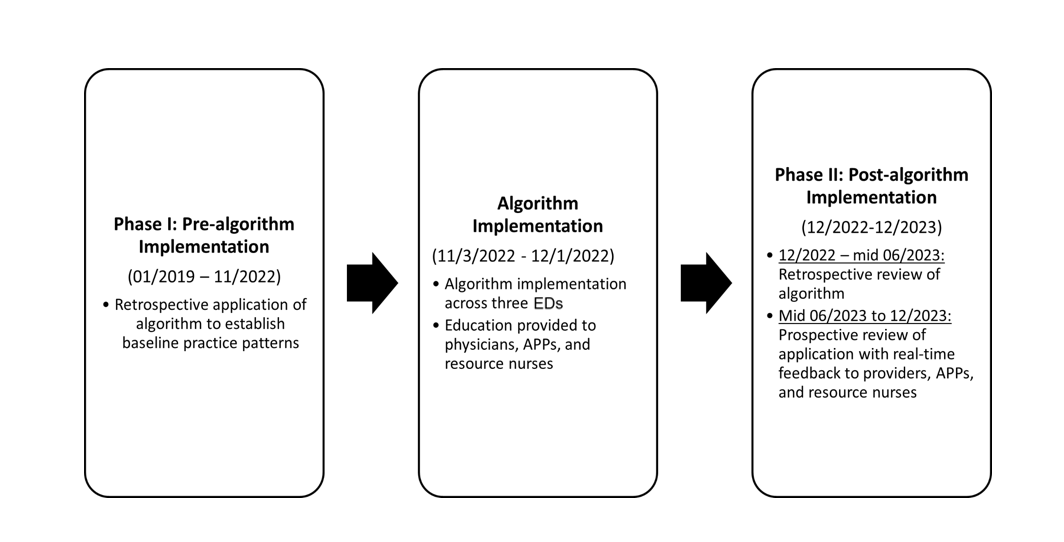


**Table S1.** Bacteria types and ED response by hospital and by pre- and post-implementation period

|  | **Academic Medical Center** | | **Community Hospital #1** | | **Community Hospital #2** | |
| --- | --- | --- | --- | --- | --- | --- |
|  | **Pre** | **Post** | **Pre** | **Post** | **Pre** | **Post** |
|  | **N = 118** | **N = 30** | **N = 40** | **N = 18** | **N = 30** | **N = 17** |
| **CoNS** | | | | | | |
| Yes | 104 (88.1%) | 30 (100.0%) | 27 (67.5%) | 14 (77.8%) | 23 (76.7%) | 14 (82.4%) |
| No | 14 (11.9%) | 0 (0.0%) | 13 (32.5%) | 4 (22.2%) | 7 (23.3%) | 3 (17.6%) |
| ***S. aureus*** | | | | | | |
| Yes | 15 (12.7%) | 0 (0.0%) | 14 (35.0%) | 4 (22.2%) | 7 (23.3%) | 2 (11.8%) |
| No | 103 (87.3%) | 30 (100.0%) | 26 (65.0%) | 14 (77.8%) | 23 (76.7%) | 15 (88.2%) |
| **Patients told to Return to ED** | | | | | | |
| Yes | 80 (67.8%) | 15 (50.0%) | 25 (62.5%) | 12 (66.7%) | 10 (33.3%) | 8 (47.1%) |
| No | 38 (32.2%) | 15 (50.0%) | 15 (37.5%) | 6 (33.3%) | 20 (66.7%) | 9 (52.9%) |
| **Patient Returned to ED** | | | | | | |
| Yes | 76 (64.4%) | 12 (40.0%) | 21 (52.5%) | 10 (55.6%) | 10 (33.3%) | 5 (29.4%) |
| No | 42 (35.6%) | 18 (60.0%) | 19 (47.5%) | 8 (44.4%) | 20 (66.7%) | 12 (70.6%) |
| **Appropriate ED Response** | | | | | | |
| Yes | 78 (66.1%) | 23 (76.7%) | 28 (70.0%) | 15 (83.3%) | 23 (76.7%) | 17 (100.0%) |
| No | 40 (33.9%) | 7 (23.3%) | 12 (30.0%) | 3 (16.7%) | 7 (23.3%) | 0 (0.0%) |
